# Serial Intervals and Household Transmission of SARS-CoV-2 Omicron Variant, South Korea, 2021

**DOI:** 10.3201/eid2803.212607

**Published:** 2022-03

**Authors:** Jin Su Song, Jihee Lee, Miyoung Kim, Hyeong Seop Jeong, Moon Su Kim, Seong Gon Kim, Han Na Yoo, Ji Joo Lee, Hye Young Lee, Sang-Eun Lee, Eun Jin Kim, Jee Eun Rhee, Il Hwan Kim, Young-Joon Park

**Affiliations:** Capital Regional Center for Disease Control and Prevention, Seoul, South Korea (J.S. Song, J. Lee, M. Kim);; Incheon Metropolitan Government, Incheon, South Korea (H.S. Jeong, M.S. Kim, S.G. Kim, H.N. Yoo);; Korea Disease Control and Prevention Agency, Cheongju, South Korea (J.J. Lee, H.Y. Lee, S.-E. Lee, E.J. Kim, J.E. Rhee, I.H. Kim, Y.-J. Park)

**Keywords:** COVID-19, 2019 novel coronavirus disease, coronavirus disease, severe acute respiratory syndrome coronavirus 2, SARS-CoV-2, viruses, respiratory infections, zoonoses, Omicron, variant of concern, South Korea

## Abstract

To clarify transmissibility of the severe acute respiratory syndrome coronavirus 2 Omicron variant, we determined serial intervals and secondary attack rates among household contacts in South Korea. Mean serial interval for 12 transmission pairs was 2.9 days, and secondary attack rate among 25 households was 50.0%, raising concern about a rapid surge in cases.

The severe acute respiratory syndrome coronavirus 2 (SARS-CoV-2) Omicron (B.1.1.529) variant of concern has been confirmed on all continents and has spread through communities around the world at unprecedented speed (*1*). Given uncertainties about current estimates of virus transmissibility, we analyzed real-life data on serial intervals for transmission pairs (time from infector symptom onset to infectee symptom onset) and secondary attack rate among household contacts, offering metrics essential for predicting epidemic size, forecasting healthcare demand, and devising effective public health interventions. Details of the epidemiologic situation with regard to importation and transmission of the Omicron variant in South Korea have been described elsewhere (*2*). We further traced a total of 76 case-patients with Omicron infection that originated from 2 persons with imported cases (75 confirmed cases and 1 suspected case) and their contacts, focusing on infector-infectee relationships and household transmission during November–December 2021.

Because of the possibility of their being exposed to other potential sources of infection at church on November 28, 2021, we excluded infectees who had visited church on that date from transmission pairs. As for the time of infection in households, we assumed that the earliest exposure occurred 2 days before symptom onset of an infector and the last exposure before isolation of the infector. To calculate serial intervals, we did not include case-patients without a clear date of symptom onset. We defined a household as a group of persons living in the same residence with a shared space. This study was conducted as a legally mandated public health investigation under the authority of the Korean Infectious Diseases Control and Prevention Act (no. 12444 and no. 13392). The study was not research that was subject to institutional review board approval; therefore, written informed consent was not required.

We identified 25 households, comprising 55 household members. Only 1 household comprised South Korea nationals; the others, foreign nationals. Of the 55 household members, 36 were confirmed to be Omicron-positive, among which secondary attack rate was 0.65 (95% CI 0.48–0.81). After we excluded the 18 household members who had visited church on November 28, 2021, the remaining 18 were confirmed to be Omicron case-patients; secondary attack rate among the 18 was 0.50 (95% CI 0.35–0.72) (Table).

**Table:** Characteristics of SARS-CoV-2 Omicron (B.1.1.529) variant of concern of index case-patients and household members, South Korea, 2021*

Household	Index case-patient					Household members†	
	Age, y/sex	Transmission route	Signs/symptoms at diagnosis	COVID-19 vaccination status (vaccine)‡		No. members, n = 36	No. confirmed cases, n = 18
1	44/M	Imported case	Cough, sputum, sore throat	Fully (mRNA-1273)		2	1
2	38/M	Contact with a confirmed case at the airport	Fever, cough, myalgia	Unvaccinated		2	1
3	33/M	Contact at church and restaurant	Fever, cough, myalgia, headache	Unvaccinated		1	1
4	39/F	Contact at church	Fever, myalgia	Partially (BNT162b)		1	0
5	31/F	Contact at church	Myalgia	Unvaccinated		0	0
6	27/F	Contact at church	Fever, sore throat	Unvaccinated		1	1
7	33/F	Contact at church	Fever, chill, cough, myalgia, headache	Unvaccinated		2	2
8	34/F	Contact at church	Sore throat	Unvaccinated		2	1
9	50/F	Contact at church	Cough, sore throat, headache	Fully (Ad26.COV2.S)		1	1
10	56/F	Contact with a friend	Cough, fatigue	Fully (Ad26.COV2.S)		0	0
11	46/M	Contact at church	Asymptomatic	Fully (mRNA-1273)		0	0
12	39/F	Contact at a restaurant	Sore throat	Unvaccinated		2	2
13	77/F	Contact at church	Cough, sore throat	Fully (BNT162b)		2	0
14	44/F	Contact at church	Sputum, sore throat	Fully (mRNA-1273)		1	1
15	6/M	Contact at a childcare center	Asymptomatic	Unvaccinated		2	0
16	31/F	Contact at church	Fever, sputum, sore throat	Unvaccinated		1	0
17	23/F	Contact with a friend	Fever, chill, cough, sputum, sore throat, myalgia, headache	Fully (mRNA-1273)		1	0
18	4/M	Contact at street	Asymptomatic	Unvaccinated		3	0
19	64/F	Contact at church	Asymptomatic	Fully (mRNA-1273)		0	0
20	67/F	Contact at church	Asymptomatic	Unvaccinated		1	1
21	34/F	Contact at church	Fever, myalgia	Unvaccinated		1	1
22	33/F	Contact at church	Sore throat, rhinorrhea	Fully (BNT162b)		2	0
23	45/F	Contact with a family member	Asymptomatic	Fully (Ad26.COV2.S)		2	1
24	2/F	Contact at playground	Fever, rhinorrhea	Unvaccinated		4	3
25	3/M	Contact at a childcare center	Asymptomatic	Unvaccinated		2	1
Total		NA	NA	NA		36	18
Attack rate		NA	NA	NA		NA	0.50

*COVID-19, coronavirus disease; SARS-CoV-2, severe acute respiratory syndrome coronavirus 2. †Household members who participated in church service on November 28, 2021, were excluded. ‡An unvaccinated person had received no COVID-19 vaccine. A partially vaccinated person had received a COVID-19 vaccine but had not completed the primary series >14 d before illness onset. A fully vaccinated person had completed the primary series of COVID-19 vaccine >14 d before illness onset; vaccinated index case-patients received BNT162b, n = 3; mRNA-1273, n = 5; or Ad26.COV2.S, n = 3.

We used 12 transmission pairs for the calculation, including 12 infectors and 19 infectees. Mean (± SD) ages were 34.2 (± 18.2) years for infectors and 32.5 (± 21.7) years for infectees. The mean incubation period of the transmission pairs was 2.5–4.3 days, and the median incubation period was 3–4 days. The mean (± SD) serial interval for the pairs was 2.9 (± 1.6) days; the median serial interval was 3.0 days (Appendix).

The estimated mean serial interval of 2.9 days for Omicron was shorter than that determined for wild-type virus and the Delta variant found in other studies conducted in South Korea (*3*,*4*). Enhanced nonpharmaceutical interventions such as rapid isolation of case-patients, as revealed by the mean time of 0.75 (range 0–4) days from symptom onset to isolation among infectors, and meticulous contact tracing during the study period may have shortened the serial interval and reduced superspreading potential, as evidenced in other research (*5*). Thus, further studies in other places or at other periods, are needed, using larger sample sizes to more accurately estimate transmission dynamics and effects of public health measures.

The household secondary attack rate that we found, factoring in vaccination status and prior infections, was substantially higher than rates for wild type virus and the Delta variant of concern previously reported in South Korea and other countries (*6*). This finding is in line with earlier reports that suggested increased household risk for transmission of Omicron variant (*7*,*8*), although enhanced isolation in conjunction with a comprehensive testing strategy for contacts of case-patients may partially inflate secondary attack rate in our study. Of note, in our study, the secondary attack rate among fully vaccinated persons is high (62.5%, 10/16), thus heightening concerns over immune escape and the possibility that Omicron may be associated with considerably reduced vaccine effectiveness. However, further studies are needed to accurately assess the relative roles of increased intrinsic transmissibility and immune escape.

Our findings with regard to Omicron transmissibility by symptomatic index case-patients supports that of a meta-analysis reporting that that secondary attack rates were higher in households with symptomatic rather than asymptomatic index case-patients (*6*). However, caution is warranted when interpreting our results because other social and demographic factors could not be properly adjusted and sample size was too small to ensure adequate statistical power. Our findings of a short serial interval among transmission pairs and a high secondary attack rate among household members adds timely real-life evidence of increased transmissibility of the Omicron variant of concern along with the potential for immune escape, thus necessitating a package of effective public health measures to mitigate the spread of Omicron in each country.

Appendix Supplemental information for transmission pairs infected with severe acute respiratory syndrome coronavirus 2 Omicron (B.1.1.529) variant of concern, South Korea, 2021.
